# Short-term milk production responses differ when balancing dietary 18-carbon fatty acids using high-oleic soybeans or a commercially available stearic acid-enriched supplement

**DOI:** 10.3168/jdsc.2026-1022

**Published:** 2026-05-15

**Authors:** Brooke A. Seelenbinder, Jair E. Parales-Girón, Alycia M. Bales, Adam L. Lock

**Affiliations:** Department of Animal Science, Michigan State University, MI 49964

## Abstract

Recent evidence suggests that dietary 16- and 18-carbon fatty acids (FA) play an important role in influencing milk fat synthesis and nutrient partitioning. However, limited research has directly compared the effects of oleic- versus stearic-based supplemental sources under controlled conditions. We evaluated the effects of supplying 18-carbon FA primarily as oleic acid (*cis*-9 C18:1) from high-oleic soybeans versus stearic acid (C18:0) from a commercially available C18:0-enriched supplement, while balancing for similar 16-carbon FA intake, on short-term production responses in mid-lactation Holstein cows. Thirty-two cows were assigned to a 2-period crossover design with two 14-d periods, with sampling during the final 4 d of each period. Treatments were diets containing either 8% of diet DM as roasted, ground high-oleic soybeans (HOS) or 2% of diet DM as a C18:0-enriched supplement (SAT). High-oleic soybeans replaced soybean-based bypass protein to maintain similar overall dietary nutrient composition. Because inclusion of the C18:0-enriched supplement also increased dietary palmitic acid (C16:0), a C16:0-enriched supplement was added to the HOS diet to balance C16:0 supply and better isolate the effects of C18:0 versus *cis*-9 C18:1. The statistical model included cow within square as a random effect, and treatment, period, and square as fixed effects. Although diets were formulated to provide similar amounts of total and 16- and 18-carbon FA, analyzed FA profiles revealed that the C18:0-enriched supplement in SAT delivered a lower 18-carbon FA supply than its formulation values suggested. Compared with SAT, HOS reduced DMI (31.2 vs. 32.0 kg/d) but increased milk yield (50.8 vs. 49.0 kg/d), milk fat (2.05 vs. 1.97 kg/d), milk protein (1.59 vs. 1.52 kg/d), and lactose (2.49 vs. 2.39 kg/d). Accordingly, HOS increased 3.5% FCM by 2.3 kg/d, and ECM increased by 2.4 kg/d. A decrease in mixed FA yield was observed with HOS (721 vs. 778 g/d), along with an increase in preformed FA yield (717 vs. 614 g/d), consistent with the greater milk fat output. Body weight gain was also higher for HOS (0.78 vs. −0.01 kg/d), although the short evaluation period limits conclusions regarding longer-term effects. Collectively, these results indicate that feeding high-oleic soybeans, which mainly provide 18-carbon FA as *cis*-9 C18:1, led to better short-term milk production and component yields than using a commercially available C18:0-enriched supplement. These findings provide further evidence that individual 18-carbon FA exert different metabolic and production effects and should not be treated as interchangeable in diet formulation. More broadly, they highlight the value of accurate nutrient composition data in feed libraries and demonstrate that high-oleic soybeans may offer a practical on-farm alternative to certain commercially available fat supplements.

## Highlights

• High-oleic soybeans increased milk yield versus the stearic acid-enriched supplement.
• High-oleic soybeans improved feed efficiency, showing greater energy-corrected milk (ECM) yield at lower DMI.
• High-oleic soybeans can provide a cost-effective alternative to certain fat supplements.

## Graphical Abstract

**Figure:**
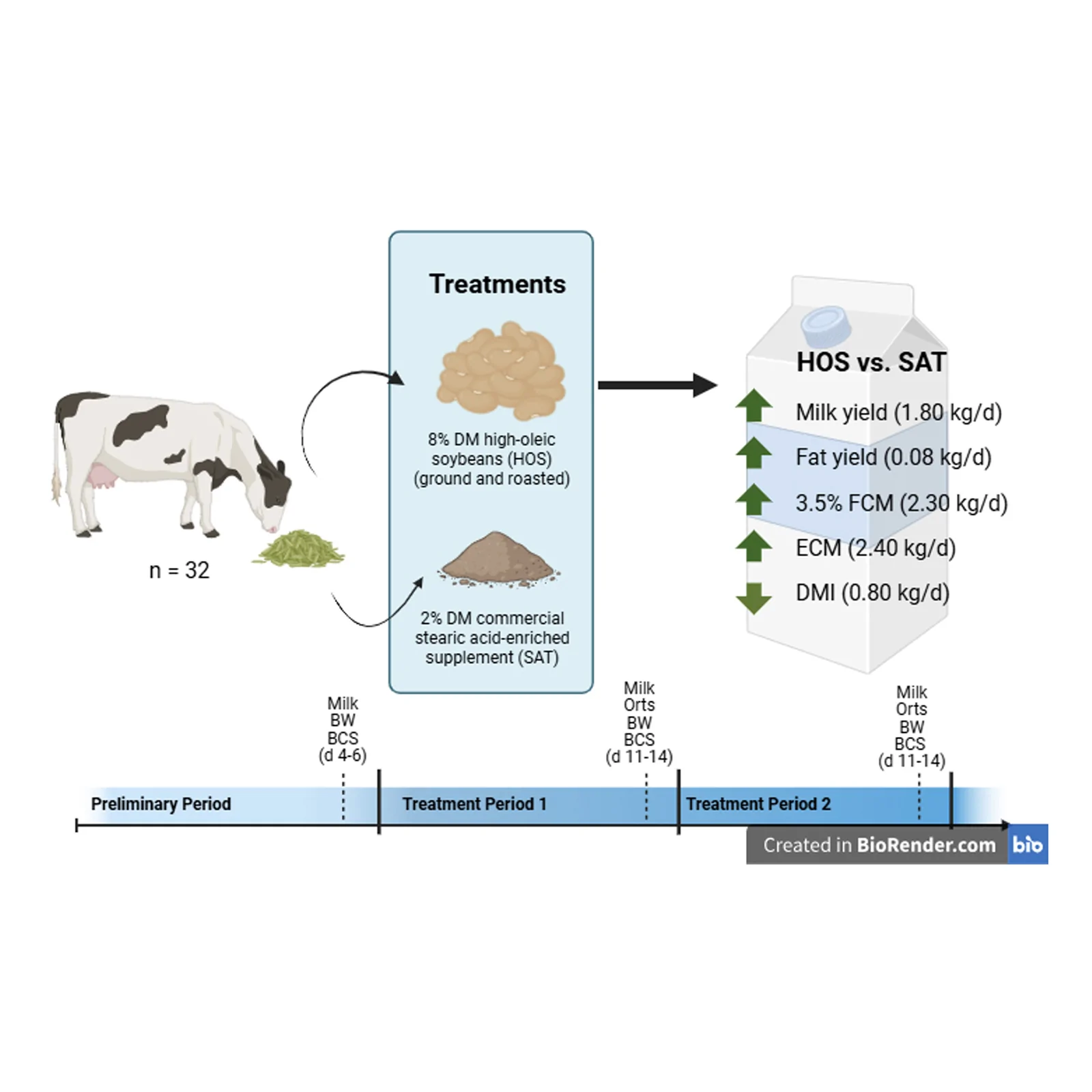
**Summary:** This study assessed the effects of balancing 18-carbon fatty acids in mid-lactation dairy cows by using either high-oleic soybeans (HOS) or a supplement enriched with stearic acid (SAT), while keeping the intake of 16-carbon fatty acids similar. In a 2-treatment crossover design, HOS increased the yields of milk and milk components as well as feed efficiency compared with SAT, despite reducing dry matter intake (DMI). Cows fed HOS also gained more body weight (BW) and shifted milk fatty acid composition toward increased preformed fatty acids. These results demonstrate that HOS is an effective on-farm alternative to stearic acid-enriched supplements, offering both production and nutritional benefits when formulating rations to optimize performance in mid-lactation cows. BCS = body condition score; FCM = fat-corrected milk.

Improving milk fat and protein yields remains a central goal in dairy production, as these components directly influence both milk's economic value and the efficiency of nutrient utilization. Dietary fatty acids (**FA**) are widely supplemented to increase energy density and support milk component synthesis, commonly through oilseeds or commercial fat supplements (Rabiee et al., 2012; Lock et al., 2025). Supplemental FA differ in digestibility and metabolic fate, and we have previously shown that palmitic (C16:0), stearic (C18:0), and oleic acids (*cis*-9 C18:1), the predominant FA in many supplements, elicit distinct production responses (dos Santos Neto et al., 2021a,b). Although mixtures of C16:0 and C18:0 have been recommended to optimize FA utilization (Loften et al., 2014), subsequent studies indicate that replacing C18:0 with either C16:0 or *cis*-9 C18:1 often increases the yields of milk and milk components—responses generally attributed to the greater digestibility and metabolic efficiency of C16:0 and *cis*-9 C18:1 compared with C18:0 (Lock et al., 2025).

High-oleic soybean cultivars, containing over 70% *cis*-9 C18:1, provide a practical on-farm source of *cis*-9 C18:1 with limited polyunsaturated FA, and recent work reports increased yields of milk and milk components when high-oleic soybeans are included in rations (Bales and Lock, 2024a,b). Heat treatment further improves soybean feeding value by increasing rumen-undegradable protein and reducing antinutritional factors (Real-Guerra et al., 2013), supporting the use of roasted high-oleic soybeans as both a fat and a protein source. In contrast, evidence indicates that dietary C18:0 is generally less digestible and that increasing its flow to the duodenum reduces total FA digestibility (Boerman et al., 2015a). This may help explain the more favorable production responses observed with *cis*-9 C18:1-rich sources (de Souza et al., 2018; Prom and Lock, 2021).

Despite the biological and practical relevance of these differences, no studies have directly compared roasted high-oleic soybeans against a commercially available C18:0-enriched supplement when diets are balanced for total FA, C16:0, and overall 18-carbon FA supply. This is an important comparison because ration formulation depends on accurate FA characterization and realistic assumptions about the relative efficiency of individual 18-carbon FA for supporting milk synthesis and nutrient partitioning. We therefore hypothesized that supplying 18-carbon FA primarily as *cis*-9 C18:1 from high-oleic soybeans would increase the yields of milk and milk components compared with supplying C18:0 from a commercially available C18:0-enriched supplement, even when diets are formulated to deliver similar total FA and C16:0 contents.

The study was conducted from January to February 2025 at the Michigan State University Dairy Cattle and Teaching Research Center (East Lansing, MI). Thirty-two multiparous, mid-lactation Holstein cows (49.7 ± 5.76 kg/d milk; 120 ± 33 DIM) were housed in tiestalls with free access to water and milked 3 times daily. Cows received a common diet without supplemental fat during an 11-d preliminary period before assignment to treatment sequence. Cows were blocked by milk yield and randomly assigned to 1 of 2 treatments in a 2-period crossover design with 2 consecutive 14-d periods. Treatments were as follows: (1) a diet containing 8% DM roasted and ground high-oleic soybeans (**HOS**) or (2) a diet containing 2% DM of a C18:0-enriched supplement (**SAT**). Cows (n = 16) in treatment sequence A received HOS in period 1 and SAT in period 2 (49.1 ± 5.16 kg milk/d; range: 40.7–61.8), whereas cows (n = 16) in treatment sequence B received SAT in period 1 and HOS in period 2 (50.3 ± 6.41 kg milk/d; range: 43.8–69.6).

Diets were formulated to provide similar concentrations of total FA, C16:0, and total 18-carbon FA using ingredient composition data from the diet formulation program's feed library (AMTS.Cattle.Professional v.4.17.0.0; Van Amburgh et al., 2015). High-oleic soybeans replaced soybean-based bypass protein to maintain similar concentrations of NDF, forage NDF, starch, CP, and RUP across diets. Because the C18:0-enriched supplement also contributed additional C16:0, a C16:0-enriched supplement was included in the HOS diet to equalize dietary C16:0 supply. Together, these adjustments allowed a more isolated evaluation of differences in 18-carbon FA. Ingredient and nutrient compositions of the experimental diets are presented in Table 1.

**Table 1:** Ingredients and nutrient compositions of treatment diets

Item	Treatment	
	SAT	HOS
Ingredient, % DM		
Corn silage	43.9	43.9
Alfalfa silage	11.9	11.9
Ground corn	15.0	15.0
Soybean meal	4.21	4.56
Lactation mix^1^	7.03	7.03
Mineral and vitamin mix^2^	1.98	1.98
DCAD supplement^3^	0.36	0.36
Soyhulls	6.09	5.90
Soybean-based bypass protein^4^	7.48	—
Roasted high-oleic soybeans^5^	—	8.58
C16:0-enriched supplement^6^	—	0.74
C18:0-enriched supplement^7^	2.03	—
Nutrient composition, % DM		
NDF	28.6	29.3
Forage NDF	21.1	21.1
Starch	27.0	27.9
CP	16.0	16.2
FA	3.45	4.03

1 Contained 39.0% soybean-based bypass protein (AminoPlus; Ag Processing Inc., Omaha, NE); 16.9% ground corn; 15.8% sodium sesquicarbonate; 12.9% calcium carbonate; 12.2% blood blend (Spectrum AgriBlue; Perdue Agribusiness, Salisbury, MD); 2.6% urea; and 0.6% rumen-protected Met (Smartamine M; Adisseo, Alpharetta, GA).

2 Contained 20.8% calcium carbonate; 16.0% calcium magnesium carbonate (MIN-AD; Min-Ad Inc., Winnemucca, NV); 14.4% white salt; 13.3% ground corn; 12.7% calcium phosphate; 9.3% magnesium oxide; 4.8% magnesium sulfate; 4.5% sodium sesquicarbonate; 2.4% selenium; 0.7% tallow; and <1% ferrous sulfate, trace mineral mix (Micro 5; Selko, Indianapolis, IN), rumen-protected Met (Smartamine M; Adisseo, Alpharetta, GA), vitamin D, vitamin E, and vitamin A.

3 Ion Plus (D&D Ingredients LLC, Delphos, OH), containing 48.5% potassium and 0.31% sodium.

4 AminoPlus (Ag Processing Inc., Omaha, NE).

5 Plenish soybeans (Pioneer; Johnston, IA). Fatty acid profile (17.5% DM of total FA) consisted of (g/100 g FA): 5.00 C16:0, 3.37 C18:0, 83.4 *cis*-9 C18:1, 4.78 *cis*-9,*cis*-12 C18:2, and 1.46 *cis*-9,*cis*-12,*cis*-15 C18:3.

6 Propalm 85 (Perdue AgriBusiness, Salisbury, MD). The supplement (99.9% total FA content) contained (g/100 g FA) 1.32 C14:0, 89.4 C16:0, 0.74 C18:0, 6.78 *cis*-9 C18:1, and 1.23 *cis*-9,*cis*-12 C18:2.

7 Energy Booster 100 (Actus Nutrition, Eden Prairie, MN). The supplement (85.9% total FA content) contained (g/100 g FA) 2.21 C14:0, 42.8 C16:0, 43.5 C18:0, 7.48 *cis*-9 C18:1, and 1.18 *cis*-9,*cis*-12 C18:2.

The high-oleic soybeans used in the experiment were processed using a flame roaster and a hammer mill. The soybeans were heated to between 143°C and 149**°**C, steeped for 30 min (Hsu and Satter, 1995), and further analyzed for roasting quality and grind size (Cumberland Valley Analytical Services, Waynesboro, PA). On average (±SD), the protein dispersion index was 9.9 ± 1.9 units, 16-h RUP (% of CP; Ross et al., 2013) was 48.0% ± 5.8%, and particle size was 818 ± 2.21 μm.

Samples of diet and orts were collected during the final 4 d of each period, composited by cow and period, and analyzed for nutrient composition (Boerman et al., 2017; Bales et al., 2024a). Milk yield was recorded at each milking, and milk samples were analyzed for fat, true protein, and lactose by mid-infrared spectroscopy (CentralStar Cooperative, Grand Ledge, MI). Milk lipid extraction and FA methyl ester preparation and quantification followed established gas chromatography procedures (Bales and Lock, 2024a). Yields of individual FA in milk fat were calculated following Benoit et al. (2024). Body weight, BW change, and BCS were collected as described by Boerman et al. (2015b).

Data were analyzed using the PROC GLIMMIX model procedure of SAS (version 9.4; SAS Institute Inc., Cary, NC). The statistical model included cow within block as a random effect, and treatment, period, and block as fixed effects. The period × treatment interaction was removed because it was not significant. Normality was assessed using box plots and normal probability plots, and homogeneity of variances was verified. Treatment effects were declared significant at *P* ≤ 0.05 and tendencies at 0.05 < *P* ≤ 0.10. Results are presented as least squares means ± SEM.

Gas chromatography analysis of dietary ingredients used during the treatment periods indicated that the commercial C18:0-enriched supplement contained lower total FA and 18-carbon FA than the amounts specified in the feed library of the diet formulation program, resulting in a lower FA content in the SAT diet than formulated. Accordingly, Table 2 presents the predicted and observed FA contents for the respective diets, along with the estimated dietary protein supply.

**Table 2:** Observed^1^ and predicted^2^ contents of FA and protein fractions of treatment diets

Item	Treatment	
	SAT	HOS
Observed FA content, % DM		
C16:0	1.02	0.99
C18:0	0.80	0.10
*cis*-9 C18:1	0.31	1.68
*cis*-9,*cis*-12 C18:2	0.97	0.99
*cis*-9,*cis*-12,*cis*-15 C18:3	0.13	0.09
Total 18-carbon	2.21	2.86
Total FA	3.45	4.03
Predicted FA content, % DM		
C16:0	1.05	1.03
C18:0	1.09	0.13
*cis*-9 C18:1	0.60	1.60
*cis*-9,*cis*-12 C18:2	1.25	1.23
*cis*-9,*cis*-12,*cis*-15 C18:3	0.22	0.23
Total 18-carbon	3.15	3.18
Total FA	4.47	4.34
Observed dietary protein		
CP, % DM	16.2	16.0
RDP, % CP	51.2	52.8
RUP, % CP	48.8	47.2
Soluble protein, % CP	32.6	33.3
MP supply, g/d	3,825	3,661
Predicted dietary protein		
CP, % DM	16.7	16.7
RDP, % CP	53.4	54.5
RUP, % CP	46.6	45.5
Soluble protein, % CP	32.1	32.6
MP supply, g/d	3,257	3,225

1 Observed dietary FA content based on FA analysis performed internally as described by Bales et al. (2024a), and protein-related nutrients based on feed ingredient analyses (Cumberland Valley Analytical Services, Waynesboro, PA) for reformulation (AMTS.Cattle.Professional v. 4.17.0.0; AMTS LLC; Van Amburgh et al., 2015).

2 Predicted dietary FA content and protein-related nutrients based on protein ingredients within the feed library of the AMTS software (AMTS.Cattle.Professional v. 4.17.0.0; AMTS LLC; Van Amburgh et al., 2015).

Production results and milk FA source are provided in Table 3. Compared with SAT, HOS reduced DMI (0.80 kg/d; *P* = 0.03) and increased yields of milk (1.8 kg/d; *P* < 0.01), milk fat (0.08 kg/d; *P* = 0.05), milk protein (0.07 kg/d; *P* = 0.01), and milk lactose (0.10 kg/d; *P* < 0.01). Consequently, HOS also increased the yields of 3.5% FCM (2.3 kg/d; *P* = 0.01) and ECM (2.4 kg/d; *P* = 0.01). On a milk-content basis, HOS decreased milk fat (0.10 percentage units; *P* < 0.01), increased lactose (0.01 percentage units; *P* = 0.01), did not affect protein (*P* = 0.98), and reduced MUN (2.1 mg/dL; *P* < 0.01). Feed efficiency improved with HOS, as indicated by a 0.12 kg/kg greater ECM/DMI (*P* < 0.01). The HOS treatment also increased BW (5 kg; *P* = 0.02) and BW gain (0.77 kg/d; *P* < 0.01) but did not affect BCS (*P* = 0.51).

**Table 3:** Dry matter intake, production responses, and summation of milk FA content and yield of cows fed treatment diets

Variable	Treatment		SEM	*P*-value
	SAT	HOS		
DMI, kg/d	32.0	31.2	0.35	0.03
Yield, kg/d				
Milk	49.0	50.8	0.57	<0.01
Fat	1.97	2.05	0.03	0.05
Protein	1.52	1.59	0.02	0.01
Lactose	2.39	2.49	0.03	<0.01
3.5% FCM^1^	52.5	54.8	0.61	0.01
ECM^2^	51.7	54.1	0.62	0.01
Content, g/100 g				
Fat	4.16	4.06	0.10	<0.01
Protein	3.15	3.15	0.04	0.98
Lactose	4.93	4.94	0.02	0.01
MUN, mg/dL	14.6	12.5	0.24	<0.01
ECM/DMI, kg/kg	1.62	1.74	0.02	<0.01
BW, kg	735	740	9.40	0.02
BW change, kg/d	−0.01	0.78	2.60	<0.01
BCS	2.88	2.87	0.03	0.51
Summation by source,^3^ g/100 g of FA				
De novo	25.5	24.6	0.23	<0.01
Mixed	41.6	37.7	0.38	<0.01
Preformed	32.9	37.7	0.35	<0.01
Summation by source, g/d				
De novo	478	469	11.4	0.35
Mixed	778	721	18.9	<0.01
Preformed	614	717	11.4	<0.01
Summation by source, mmol/d				
De novo	2,772	2,732	65.8	0.45
Mixed	3,034	2,811	73.8	<0.01
Preformed	2,169	2,532	40.5	<0.01

1 Fat-corrected milk: 3.5% FCM = (0.4324 × kg milk) + (16.216 × kg milk fat).

2 Energy-corrected milk: ECM = (0.327 × kg milk) + (12.95 × kg milk fat) + (7.20 × kg milk protein).

3 De novo FA originate from mammary de novo synthesis (<16 carbons); preformed FA originate from extraction from plasma (>16 carbons); and mixed FA originate from both sources (C16:0 plus *cis*-9 C16:1).

Milk FA sources differed between treatments. The HOS treatment decreased the proportion of de novo and mixed FA and increased preformed FA (all *P* < 0.01). On a yield basis, HOS reduced mixed FA and increased preformed FA (−57 and +103 g/d, respectively; both *P* < 0.01), whereas de novo FA yield was unchanged. On a molar basis (mmol/d), de novo FA were not affected (*P* = 0.45), whereas HOS reduced mixed FA and increased preformed FA (223 and 363 mmol/d, respectively; *P* < 0.01).

Recent research has renewed interest in using oilseeds such as whole cottonseed (Bales et al., 2024b; Adeniji et al., 2025) and high-oleic soybeans (Bales and Lock, 2024a,b) in dairy cow diets as alternatives to commercial fat supplements. We are also gaining deeper insight into how individual FA and blends of FA affect the performance of high-producing dairy cows (Lock et al., 2025). To our knowledge, no studies have directly compared roasted high-oleic soybeans with a commercially available C18:0-enriched supplement while balancing diets for total FA, C16:0, and 18-carbon FA supply.

Interpretation of milk FA and production responses must account for differences between the formulated and analyzed FA composition of the experimental diets. Although the diets were formulated to provide similar total FA and 18-carbon FA supply, analysis revealed that the C18:0-enriched supplement delivered less total FA and total 18-carbon FA than expected based on feed library values. Consequently, cows fed HOS consumed more total FA and more 18-carbon FA than cows fed SAT, and therefore differences in the yields of milk and milk components are explainable, at least in part, by differences in long-chain FA intake rather than providing direct evidence for an effect of FA saturation status alone.

When diets were formulated to contain similar amounts of 18-carbon FA and predicted MP supply, feeding high-oleic soybeans, which supplied *cis*-9 C18:1, supported greater yields of milk and milk components than supplying C18:0 from a fat supplement, despite slightly lower DMI and observed MP supply. This overall pattern suggests that cows fed high-oleic soybeans captured and utilized dietary energy more efficiently than those receiving the C18:0-enriched supplement. These production differences were accompanied by a shift toward greater secretion of preformed FA in milk fat, reflecting changes in the supply and metabolism of 18-carbon FA. Although unsaturated FA such as *cis*-9 C18:1 are extensively biohydrogenated in the rumen (Maia et al., 2010; Hanno et al., 2024), a notable amount remains unconverted. For SAT and HOS diets with *cis*-9 C18:1 intakes of 99.2 and 524 g/d, and assuming 30% to 60% biohydrogenation for oilseeds (Jenkins and Bridges, 2007; Barletta et al., 2016), ∼30 to 60 and 157 to 314 g/d of *cis*-9 C18:1 would reach the intestine in cows fed SAT and HOS, respectively. If all of the *cis*-9 C18:1 that undergoes biohydrogenation is converted to C18:0, intestinal C18:0 flow in cows fed HOS would increase by 210 to 367 g/d, close to the 256 g/d intake of C18:0 observed when cows received the SAT diet. Because C16:0 was similar across diets, production differences are consistent with differences in C18:0 and *cis*-9 C18:1 supply. Overall, despite substantial ruminal biohydrogenation of dietary *cis*-9 C18:1 to C18:0, these findings are consistent with an advantage of feeding *cis*-9 C18:1 rather than C18:0, in part because not all unsaturated FA are converted to saturated FA.

Previous studies support the benefits of providing dietary 18-carbon FA as *cis*-9 C18:1 rather than C18:0, which depresses FA digestibility (Boerman et al., 2017; dos Santos Neto et al., 2021a), whereas *cis*-9 C18:1 increases it (de Souza et al., 2018; Prom et al., 2021). Boerman et al. (2015a) found that higher duodenal flow of C18:0 decreased the digestibility of 16-carbon, 18-carbon, and total FA. Additionally, *cis*-9 C18:1 can reduce inflammation, lower oxidative stress, and enhance mitochondrial bioenergetics (Abou-Rjeileh et al., 2025a), which may improve energy use efficiency and partitioning in dairy cows. Improved digestibility with HOS, together with enhanced energy metabolism, likely contributed to the increase in milk yield observed here. Future studies would benefit from determining differences in nutrient digestibility across similar treatments.

With HOS, the milk fat response was due to higher preformed FA yield, lower mixed FA yield, and unchanged de novo FA yield. This shift aligns with the substantially greater *cis*-9 C18:1 intake from HOS and is consistent with evidence that *cis*-9 C18:1-rich sources are digested and absorbed more efficiently than C18:0 (de Souza et al., 2018; Prom and Lock, 2021). The resulting higher contribution of absorbed 18-carbon FA to milk fat, together with increased milk and component yields, indicates that 18-carbon FA were more available for secretion in milk fat, consistent with the greater intake and utilization of 18-carbon FA when cows were fed HOS rather than SAT. Furthermore, the combination of lower DMI and greater ECM yield with HOS suggests enhanced dietary energy capture and utilization. This pattern, also observed in previous studies with *cis*-9 C18:1-rich supplements supporting higher milk production without increasing intake (Burch et al., 2021; Prom and Lock, 2021), demonstrates that providing a greater *cis*-9 C18:1 supply supports more efficient nutrient use than relying predominantly on dietary C18:0. Responses to *cis*-9 C18:1 vary with production level and stage of lactation, with prior work demonstrating the greatest benefits in high-producing cows (≈45–60 kg/d), consistent with the production level of cows in the present study (de Souza et al., 2019; Western et al., 2020; Burch et al., 2021). Further evaluation during early lactation is also warranted based on previously reported improvements in intake, energy balance, and milk production when *cis*-9 C18:1 was supplied during this period (de Souza et al., 2021a, b).

Body weight gain was also greater for cows receiving HOS. Given the short 14-d treatment periods and the combination of lower DMI and greater milk energy output, BW differences should be interpreted cautiously; although some contribution of true tissue accretion cannot be excluded based on previous responses to *cis*-9 C18:1 and the greater dietary energy density of the HOS diet, short-term BW changes likely also reflect transient differences in rumen fill or gut contents rather than sustained whole-body energy accretion. Nevertheless, the direction of the BW response is consistent with previous work showing that *cis*-9 C18:1 can affect nutrient partitioning and reduce BW loss or promote BW gain in lactating cows (de Souza et al., 2019; Hu et al., 2024). Increasing dietary *cis*-9 C18:1 has also been positively related to energy partitioning toward body reserves (Lock et al., 2025). Additional mechanistic insight comes from recent work demonstrating that *cis*-9 C18:1 enhances mitochondrial function in adipose tissue by upregulating genes and proteins involved in lipogenesis and mitochondrial biogenesis, while also improving systemic insulin sensitivity (Abou-Rjeileh et al., 2025a,b). Longer-term studies are needed to determine whether the BW advantage persists across extended feeding durations.

Although the diets were designed to provide similar amounts of 18-carbon FA, gas chromatography analysis revealed that the commercial C18:0-enriched supplement contained a lower total FA content than the amounts reported in the diet formulation program's feed library. Due to this discrepancy, intakes differed between SAT and HOS for 16-carbon FA (326 vs. 309 g/d), 18-carbon FA (707 vs. 892 g/d), and total FA (1,104 vs. 1,257 g/d). Accordingly, the interpretation of treatment differences must also consider the discrepancy between formulated and analyzed FA compositions. Given that the SAT diet delivered less 18-carbon FA than intended, the advantage of HOS likely reflects both the metabolic benefits of *cis*-9 C18:1 and the greater actual supply of 18-carbon FA. The greater FA intake in HOS may have contributed to the observed responses, highlighting the importance of accurate FA characterization when formulating experimental diets. Further research is warranted to enhance these findings by more precisely aligning FA supplies across comparable treatments. The difference between C18:0-enriched supplement values in the diet formulation program's feed library and those found through gas chromatography is significant for commercial dairy diet formulation. Nutritionists and producers depend on accurate feed library data for diet formulation, as inaccuracies can significantly influence milk production and overall farm economics.

From a practical standpoint, these findings have several important implications. First, roasted high-oleic soybeans represent a viable on-farm source of both FA and protein, consistent with recent studies demonstrating improved yields of milk and milk components when feeding high-oleic soybeans (Bales and Lock, 2024a,b). Second, the discrepancy in values between the diet formulation program's feed library and analyzed FA content for the C18:0-enriched supplement illustrates how inaccuracies in ingredient FA composition can alter actual FA supply and should be considered in ration formulation. Third, the shift toward greater yield of milk and milk components and BW gain when feeding HOS is consistent with previously reported metabolic effects of *cis*-9 C18:1 and provides a practical indicator that dietary FA supply is affecting metabolism. Fourth, although *cis*-9 C18:1 is extensively biohydrogenated to C18:0 in the rumen, these results are consistent with an advantage of feeding *cis*-9 C18:1 over directly supplying C18:0, in part because not all unsaturated FA are converted to saturated FA. The improved milk and component yields and enhanced feed efficiency observed with HOS suggest that even partial escape of *cis*-9 C18:1 from ruminal biohydrogenation, or differences in digestibility and absorption, are associated with meaningful gains in cow performance. Additional long-term research is required to investigate these differences more thoroughly.

In summary, supplying 18-carbon FA primarily as *cis*-9 C18:1 from high-oleic soybeans, while balancing for similar 16-carbon FA intake, increased the yields of milk and milk components, improved feed efficiency, and shifted milk FA composition toward greater secretion of preformed FA compared with supplying C18:0 from a C18:0-enriched supplement. Although part of this advantage likely reflects the greater actual supply of 18-carbon FA with HOS, the direction and magnitude of responses reinforce the practical value of high-oleic soybeans as an alternative supplemental fat and protein source. Longer-term studies evaluating nutrient digestibility, energy partitioning, and precisely matched 18-carbon FA supply will help refine these conclusions. Collectively, these results highlight the importance of considering individual 18-carbon FA, rather than grouping them together, when formulating diets, and emphasize the need for accurate nutrient composition data in diet formulation program feed libraries.

## References

[bib1] Abou-Rjeileh U., Gouveia K., Lock A.L., Contreras G.A. (2025). Graduate Student Literature Review: Precision nutrition meets cellular insight—The mechanistic role of oleic acid in dairy cow metabolism. J. Dairy Sci..

[bib2] Abou-Rjeileh U., Lock A.L., Contreras G.A. (2025). Oleic acid promotes lipid accumulation in bovine adipocytes: The role of peroxisome proliferator-activated receptor alpha (PPARα) signaling. Animal.

[bib3] Adeniji Y.A., Bomberger R., Goodall S.R., Hristov A.N., Stefenoni H.A., Harvatine K.J. (2025). Effect of increasing dietary fat by feeding 15% whole cottonseed on milk production, total-tract digestibility, and methane emission in dairy cows. J. Dairy Sci..

[bib4] Bales A.M., Cinzori M.E., Lock A.L. (2024). Increasing palmitic acid and reducing stearic acid content of supplemental fatty acid blends improves production performance of mid-lactation dairy cows. J. Dairy Sci..

[bib5] Bales A.M., dos Santos Neto J.M., Lock A.L. (2024). Effect of increasing dietary inclusion of whole cottonseed on nutrient digestibility and milk production of high-producing dairy cows. J. Dairy Sci..

[bib6] Bales A.M., Lock A.L. (2024). Effects of increasing dietary inclusion of high oleic acid soybeans on milk production of high-producing dairy cows. J. Dairy Sci..

[bib7] Bales A.M., Lock A.L. (2024). Effects of raw and roasted high oleic soybeans on milk production of high-producing dairy cows. J. Dairy Sci..

[bib8] Barletta R.V., Gandra J.R., Bettero V.P., Araújo C.E., Del Valle T.A., Almeida G.F., Ferreira de Jesus E., Mingoti R.D., Benevento B.C., Freitas Júnior J.E., Rennó F.P. (2016). Ruminal biohydrogenation and abomasal flow of fatty acids in lactating cows: Oilseed provides ruminal protection for fatty acids. Anim. Feed Sci. Technol..

[bib9] Benoit A.C., dos Santos Neto J.M., Lock A.L. (2024). Mammary gland responses to altering the supply of de novo fatty acid substrates and preformed fatty acids on the yields of milk components and milk fatty acids. J. Dairy Sci..

[bib10] Boerman J.P., de Souza J., Lock A.L. (2017). Milk production and nutrient digestibility responses to increasing levels of stearic acid supplementation of dairy cows. J. Dairy Sci..

[bib11] Boerman J.P., Firkins J.L., St-Pierre N.R., Lock A.L. (2015). Intestinal digestibility of long-chain fatty acids in lactating dairy cows: A meta-analysis and meta-regression. J. Dairy Sci..

[bib12] Boerman J.P., Potts S.B., VandeHaar M.J., Lock A.L. (2015). Effects of partly replacing dietary starch with fiber and fat on milk production and energy partitioning. J. Dairy Sci..

[bib13] Burch A.M., Pineda A., Lock A.L. (2021). Effect of palmitic acid-enriched supplements containing stearic or oleic acid on nutrient digestibility and milk production of low- and high-producing dairy cows. J. Dairy Sci..

[bib14] de Souza J., Preseault C.L., Lock A.L. (2018). Altering the ratio of dietary palmitic, stearic, and oleic acids in diets with or without whole cottonseed affects nutrient digestibility, energy partitioning, and production responses of dairy cows. J. Dairy Sci..

[bib15] de Souza J., Prom C.M., Lock A.L. (2021). Altering the ratio of dietary palmitic and oleic acids affects nutrient digestibility, metabolism, and energy balance during the immediate postpartum in dairy cows. J. Dairy Sci..

[bib16] de Souza J., Prom C.M., Lock A.L. (2021). Altering the ratio of dietary palmitic and oleic acids affects production responses during the immediate postpartum and carryover periods in dairy cows. J. Dairy Sci..

[bib17] de Souza J., St-Pierre N.R., Lock A.L. (2019). Altering the ratio of dietary C16:0 and *cis*-9 C18:1 interacts with production level in dairy cows: Effects on production responses and energy partitioning. J. Dairy Sci..

[bib18] dos Santos Neto J.M., de Souza J., Lock A.L. (2021). Nutrient digestibility and production responses of lactating dairy cows when saturated free fatty acid supplements are included in diets: A meta-analysis. J. Dairy Sci..

[bib19] dos Santos Neto J.M., de Souza J., Lock A.L. (2021). Effects of calcium salts of palm fatty acids on nutrient digestibility and production responses of lactating dairy cows: A meta-analysis and meta-regression. J. Dairy Sci..

[bib20] Hanno S.L., Hurst A.M., Weaver K., Richards A.T., Montes M.E., Boerman J.P. (2024). High oleic soybean oil maintains milk fat and increases apparent total-tract fat digestibility and fat deposition in lactating dairy cows. JDS Commun..

[bib21] Hsu J.T., Satter L.D. (1995). Procedures for measuring the quality of heat-treated soybeans. J. Dairy Sci..

[bib22] Hu L., Shen Y., Zhang H., Ma N., Li Y., Xu H., Wang M., Chen P., Guo G., Cao Y., Gao Y., Li J. (2024). Effects of dietary palmitic acid and oleic acid ratio on milk production, nutrient digestibility, blood metabolites, and milk fatty acid profile of lactating dairy cows. J. Dairy Sci..

[bib23] Jenkins T.C., Bridges W.C. (2007). Protection of fatty acids against ruminal biohydrogenation in cattle. Eur. J. Lipid Sci. Technol..

[bib24] Lock A.L., dos Santos Neto J.M., de Souza J. (2025). Invited review: Moving from dietary fat to fatty acids—New insights into how fatty acids affect digestibility, metabolism, and performance in dairy cows. J. Dairy Sci..

[bib25] Loften J.R., Linn J.G., Drackley J.K., Jenkins T.C., Soderholm C.G., Kertz A.F. (2014). Invited review: Palmitic and stearic acid metabolism in lactating dairy cows. J. Dairy Sci..

[bib26] Maia M.R., Chaudhary L.C., Bestwick C.S., Richardson A.J., McKain N., Larson T.R., Graham I.A., Wallace R.J. (2010). Toxicity of unsaturated fatty acids to the biohydrogenating ruminal bacterium, *Butyrivibrio fibrisolvens.*. BMC Microbiol..

[bib27] Prom C.M., Lock A.L. (2021). Replacing stearic acid with oleic acid in supplemental fat blends improves fatty acid digestibility of lactating dairy cows. J. Dairy Sci..

[bib28] Prom C.M., dos Santos Neto J.M., Newbold J.R., Lock A.L. (2021). Abomasal infusion of oleic acid increases fatty acid digestibility and plasma insulin of lactating dairy cows. J. Dairy Sci..

[bib29] Rabiee A.R., Breinhild K., Scott W., Golder H.M., Block E., Lean I.J. (2012). Effect of fat additions to diets of dairy cattle on milk production and components: A meta-analysis and meta-regression. J. Dairy Sci..

[bib30] Real-Guerra R., Staniscuaski F., Regina C. (2013). Soybean urease: Over a hundred years of knowledge. A Comprehensive Survey of International Soybean Research—Genetics, Physiology, Agronomy and Nitrogen Relationships. J. E. Board, ed. InTech Open..

[bib31] Ross, D. A., M. Gutierrez-Botaro, and M. E. Van Amburgh. 2013. Development of an in vitro intestinal digestibility assay for ruminant feeds. Pages 190–202 in Proc. Cornell Nutr. Conf., 2013, Syracuse, NY. Cornell University.

[bib32] Van Amburgh M.E., Collao-Saenz E.A., Higgs R.J., Ross D.A., Recktenwald E.B., Raffrenato E., Chase L.E., Overton T.R., Mills J.K., Foskolos A. (2015). The Cornell Net Carbohydrate and Protein System: Updates to the model and evaluation of version 6.5. J. Dairy Sci..

[bib33] Western M.M., de Souza J., Lock A.L. (2020). Milk production responses to altering the dietary ratio of palmitic and oleic acids varies with production level in dairy cows. J. Dairy Sci..

